# Vibration mitigation of an MDoF system subjected to stochastic loading by means of hysteretic nonlinear locally resonant metamaterials

**DOI:** 10.1038/s41598-021-88984-0

**Published:** 2021-05-06

**Authors:** Francesco Basone, Oreste S. Bursi, Fabrizio Aloschi, Günter Fischbach

**Affiliations:** 1 Engineering and Architecture Faculty, University of Enna “Kore”, Viale delle Olimpiadi, 94100 Enna, Italy; 2 Department of Civil, Environmental and Mechanical Engineering, University of Trento, Via Mesiano 77, 38123 Trento, Italy; 3 IGF - Ingenieurgesellschaft Dr. Ing. Fischbach mbH, An der Vogelrute 2, 50374 Erftstadt-Lechenich, Germany

**Keywords:** Civil engineering, Mechanical engineering

## Abstract

In this paper, we intend to mitigate absolute accelerations and displacements in the low-frequency regime of multiple-degrees-of-freedom fuel storage tanks subjected to stochastic seismic excitations. Therefore, we propose to optimize a finite locally resonant metafoundation equipped with massive resonators and fully nonlinear hysteretic devices. The optimization process takes into account the stochastic nature of seismic records in the stationary frequency domain; the records are modelled with the power spectral density S_0_ and modified with a Kanai–Tajimi filter. Moreover, the massive superstructure of a fuel storage tank is also considered in the optimization procedure. To optimize the nonlinear behaviour of dampers, we use a Bouc–Wen hysteretic model; the relevant nonlinear differential equations are reduced to a system of linear equations through the stochastic equivalent linearization technique. The optimized system is successively verified against natural seismic records by means of nonlinear transient time history analyses. Finally, we determine the dispersion relations for the relevant periodic metafoundation.

## Introduction

**Figure 1:**
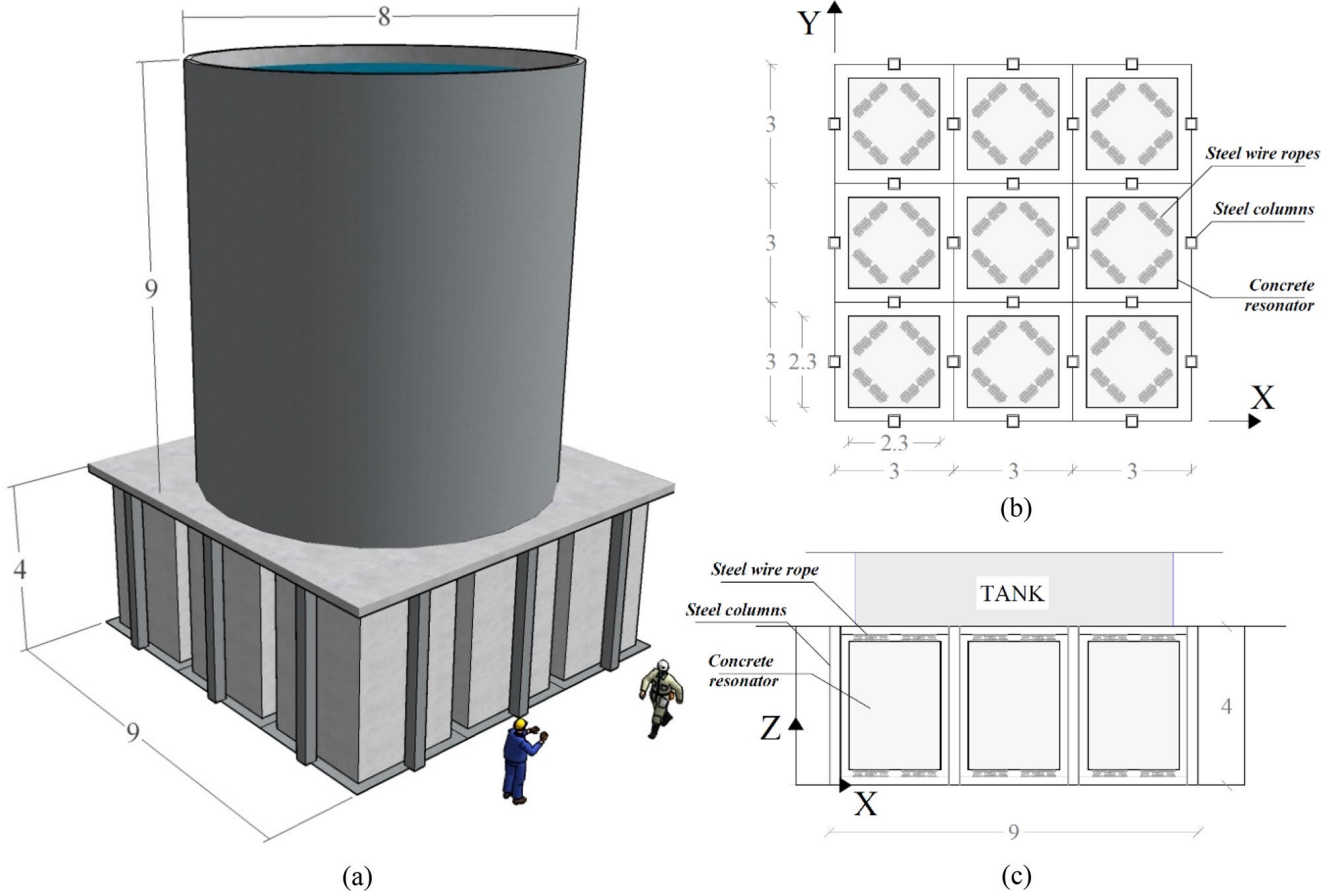
Coupled foundation—tank system: (**a**) 3D view, (**b**) layout of the unit cells and (**c**) cross section of the metafoundation. Dimensions are in m.

Within linear metamaterials, a new category of applications of phononic—or periodic—structures as alternatives to classic seismic isolators for earthquake mitigation has received growing interest^1–4^. The increasing popularity of these structures resides in the possibility of exploiting the advantages of locally resonant acoustic metamaterials (LRAMs) due to their ability to attenuate low‐frequency waves by means of unit cells much smaller than the seismic wavelength of the desired frequency region. The most common isolation solutions use lead‐rubber bearings or spherical bearing devices, which are quite effective for the horizontal components of earthquakes. Nonetheless, these solutions require two strong floors, exert a very high stiffness against the vertical component of an earthquake, and are quite ineffective for large structures subjected to rocking^5^. To reduce the seismic responses of superstructures, Cheng and Shi^3^ and Casablanca et al.^4^ studied periodic and finite locally resonant foundations. Although good response reduction results were obtained, neither of the proposed periodic systems were designed for gravity and/or seismic load combinations. Furthermore, the authors did not consider the feedback forces from the superstructures to the metafoundations. To overcome these drawbacks, we proposed a finite lattice LRAM, the so-called metafoundation, for the seismic protection of multiple-degrees-of-freedom (MDoF) systems, i.e., storage tanks^2,6,7^. The relevant coupled metafoundation-tank system is depicted in Fig. 1a.

**Figure 2:**
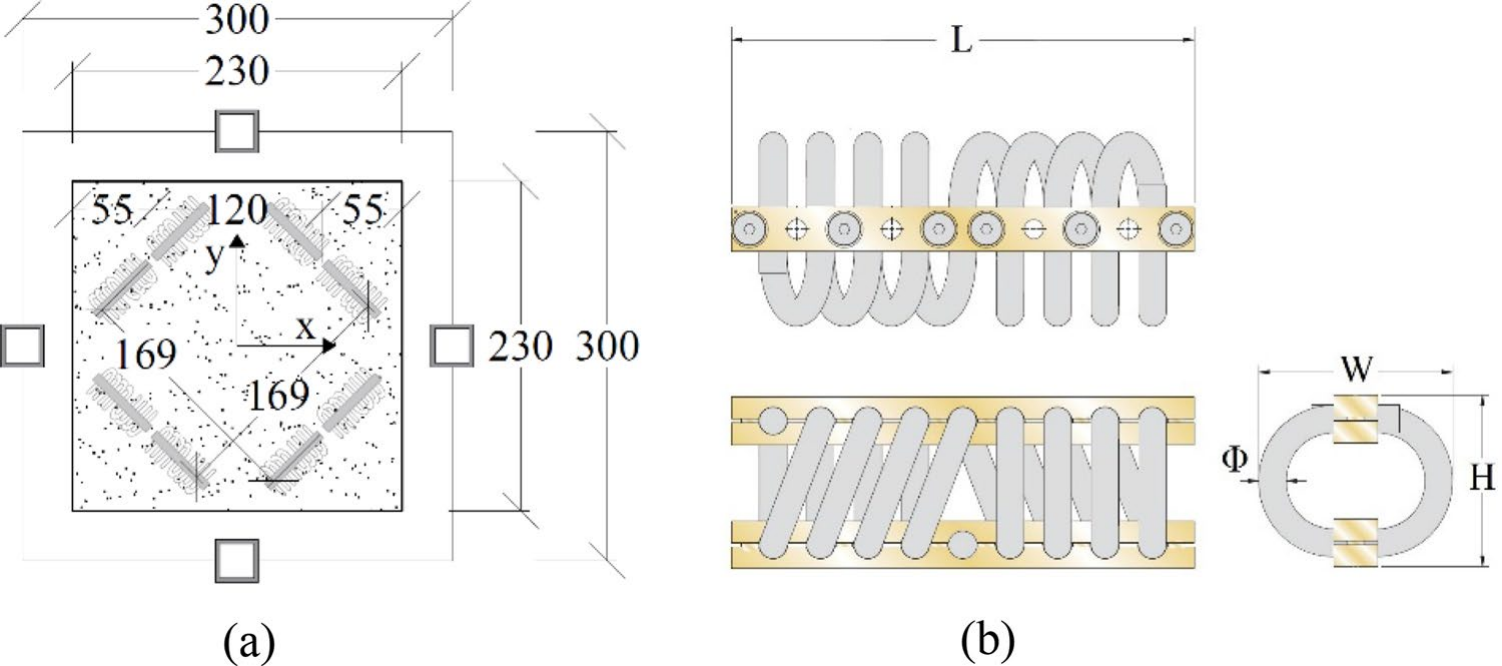
(**a**) Configuration of a single unit cell equipped with steel wire ropes; (**b**) details of a single wire rope. Dimensions are in cm.

The foundation consists of flexible steel columns and concrete slabs that define the primary load-bearing structure, while massive concrete masses are considered resonators. The construction site is Priolo Gargallo in Sicily, Italy. The site is characterized by a peak ground acceleration (PGA) of 0.56 g for a return period T_R_ = 2475 years. Therefore, a linear elastic design according to the Italian code^8^ was carried out; the resulting minimal column stiffnesses allow the metafoundation to remain undamaged following safe shutdown earthquakes (SSEs). As a result, the columns have a total height of 4 m with hollow cross-sectional dimensions of 300 × 300 mm and a thickness of 30 mm. A 3D sketch of the coupled metafoundation-tank system shows that the foundation structure is composed of a finite number of 9-unit cells. Square hollow-section steel columns support the concrete slab and provide the lateral stiffness of the metafoundation. Each unit cell includes a massive concrete cube linked to the foundation (see Fig. 1b, c) by means of wire ropes, as depicted in Fig. 2.

Nonetheless, two basic issues remain unresolved: the optimization of structural devices, i.e., springs and/or dampers, operating in the nonlinear regime within finite lattices and the inclusion of the stochastic nature of the seismic input characterized by a large random uncertainty.

With regard to the first issue, i.e., the selection of proper hysteretic dampers, Basone et al.^6^ suggested the use of wire ropes, which are simple devices able to both effectively suspend the concrete resonators inside the foundation and allow motion in all three main directions. Therefore, the behaviour of wire ropes is quite complex, and the characterization of their nonlinear properties is difficult^9,10^. More precisely, the mechanical flexibility of wire ropes provides good isolation properties, and the sliding friction between the intertwined cables results in high dissipative capabilities. As a result, these devices can achieve equivalent damping ratios of 15–20% with low production and maintenance costs. Their hysteretic behaviour can be reproduced with the well-known Bouc–Wen model^9–11^. This model is quite popular because it can describe the behaviour of a nonlinear hysteretic system with a compact first-order differential equation^12^.

If we consider the aforementioned devices in periodic systems, the analysis of nonlinear metamaterials is still very challenging^13,14^. For instance, from a perturbation approach specifically applied to weakly nonlinear periodic chains^15^, the following topics emerge: (1) the solutions to the nonlinear wave equations are amplitude dependent; (2) the wave amplitudes influence their own propagation characteristics, the so-called self-action; and (3) the analysis methods in the presence of self-action often do not trace all solutions when more than one dominant component is involved. Nonetheless, several researchers have devoted considerable effort to improving our understanding of nonlinear metamaterial-based systems. For instance, to reduce wave transmission in both ultralow and ultrabroad bands with band gaps and chaotic bands, Fang et al.^16^ developed both a theoretical approach and an experimental validation to conceive nonlinear acoustic metamaterials (NAMs). Based on a new mechanism for wave mitigation and control consisting of the nonlinear interaction between propagating and evanescent waves, Zega et al.^17^ recently presented experimental proof of the appearance of a subharmonic transmission attenuation zone due to an energy exchange induced by autoparametric resonance. In contrast, Gupta et al.^18^ explored a wide range of nonlinear mechanical behaviours that can be generated from the same lattice material by changing the building block into a dome-shaped structure. In particular, they proposed a novel hourglass-shaped lattice metastructure that takes advantage of the combination of two oppositely oriented coaxial domes.

With regard to the second issue, i.e., the stochastic nature of the seismic input and the subsequent stochastic response analysis of hysteretic systems, an abundance of literature is available^12,19,20^. In this respect, the equivalent (stochastic) linearization technique (ELT)^12,19^, based on a non-Gaussian probability density function, is viable because it can be extended (in a relatively straightforward manner) to MDoF systems. Socha and Pawleta^21^ discussed the advantages and disadvantages of this technique.

In summary, to achieve the best performance of a finite locally resonant metafoundation, the following objectives are pursued hereinafter: (1) the optimization of the nonlinear behaviour of wire ropes reproduced with hysteretic Bouc–Wen models and (2) the application of the ELT to fully nonlinear devices taking into account the stochastic nature of the seismic input in both the frequency domain and the time domain.

**Figure 3:**
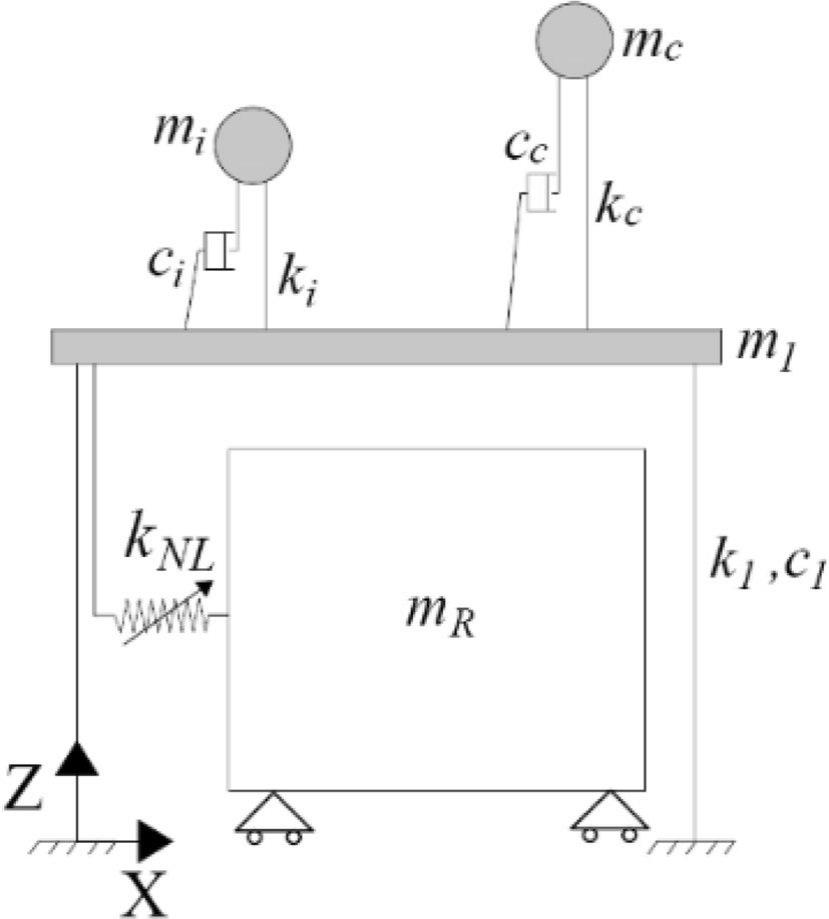
Metafoundation-tank coupled system model: condensed mass system (CMS).

The superstructure is composed of a slender fuel storage tank and its equivalent 2D lumped mass model^22^ (see Figs. 1a and 3). More precisely, the slender tank was part of an existing plant, i.e., tank #23 or #24 of Case Study #1, analysed in a European research project^2,7^. Housner’s model^23^ is adopted to simulate the hydrodynamic response of liquid containers. This model allows us to associate the inertial force of the liquid with two different masses: the impulsive mass and the convective mass. The tank response is reduced to the contributions of the two main impulsive and convective modes, and the tank wall thickness is taken into account. The tank’s liquid content exhibits axial symmetry, which is sufficient to analyse the dynamics in one direction. However, each resonator can vibrate in all three (X, Y and Z) directions.

With regard to the metafoundation, it is designed to remain undamaged for an active seismic site located in Priolo Gargallo, Italy. Considering a consistent seismic input for linear/nonlinear time history analyses, a set of natural earthquakes that correspond to SSE events are selected from Italian and European databases and fitted on average to the uniform hazard spectrum (UHS) of Priolo Gargallo.

To take the stochastic nature of the seismic input into account, the computations are carried out in the frequency domain, and because the analysis of nonlinear periodic systems entails the aforementioned difficulties^15^, the ELT is adopted for the Bouc–Wen model. Therefore, an average power spectral density (PSD) function of the accelerograms is evaluated. The resulting PSD function is filtered with a Kanai–Tajimi filter^24^ modified by Clough and Penzien^25^ and subsequently adopted in the optimization procedure. The resulting optimized metafoundation is then verified through nonlinear time history analyses (THAs) of the coupled system subjected to the aforementioned ground motions.

## Methods

### Nonlinear metafoundation system modelling

To address simpler coupled systems and to benefit from the optimization considering different stiffness and damping values, the condensed mass system (CMS) shown in Fig. 3 is considered. Through an exact dynamic condensation of both the masses and stiffnesses along X and Y directions we can analyse the CMS as a 2D system. This dynamic condensation does not lead to errors since all resonators in both the X and the Y directions are assumed to be endowed with the same mass and stiffness (see Fig. 1b). The CMS is depicted in Fig. 3, in which the dynamic characteristics of the system are reported as well. Herein, *m*_*i*_, *c*_*i*_ and *k*_*i*_ represent the mass, stiffness and damping coefficients, respectively, of the impulsive mass of the superstructure-tank system, while *m*_*c*_, *c*_*c*_ and *k*_*c*_ represent the mass, stiffness and damping coefficients of the relevant convective mass.

The following system of equations of motion (EOMs) describes the dynamics of the aforementioned metafoundation-tank coupled system:

1 $${\mathbf{M}}\;{\mathbf{\ddot{u}}}({\text{t}}) + \;{\mathbf{C}}\;{\dot{\mathbf{u}}}({\text{t}}) + \;{\mathbf{K}}\;{\mathbf{u}}({\text{t}})\; + {\rm u}_{\rm y} {\mathbf{K}}_{{}}^{{{\text{NL}}}} {\mathbf{z}}({\text{t}})\; = \;{\mathbf{F}}\;({\text{t}})$$

where **M**, **C** and **K** are the mass, damping and stiffness matrices, respectively; **K**^NL^ defines the component of the stiffness matrix that contains the terms (1 − *α*_*n*_) *k*_*n*_ introduced later; **z**(t) defines the vector that contains the components *z*_*n*_*(t)* of the *n*th resonator modelled in the next subsection; and *u*_*y*_ sets the yielding displacement of the device. Therefore, Eq. (1) is a nonlinear system of EOMs due to the presence of *u*_*y*_ **K**NL **z**(t). The vector **u**(t) indicates the displacement vector, whilst single and double dots represent single and double derivatives with respect to (w.r.t.) time, respectively. Furthermore, **F**(t) =  − **M τ** ${\ddot{\text{u}}}_{{\text{g}}}$(t) represents the forcing vector, where *τ* is the mass influence vector and *ü*_*g*_*(t)* represents the ground acceleration.

To evaluate the dynamic properties of the CMS, the system equivalent reduction expansion procedure (SEREP) is adopted. This method allows the modal vectors of the CMS to be reduced^26^; therefore, the convective mode and the relevant DoFs of the tank can be eliminated from the full set of ‘n’ DoFs, while the effects on the lower ‘a’ modes can be retained. More precisely, the SEREP technique is based on the following transformation:

2 $${\mathbf{u}}_{n} = {\mathbf{T}}\;{\mathbf{u}}_{a}$$

where **T** = **Φ**_n_ **Φ**_a_^g^ is the transformation matrix, **Φ**n is the modal matrix of the original system, and **Φ**_a_^g^ is the generalized inverse of the modal matrix of the active/reduced system. More precisely, **Φ**_a_^g^ can be evaluated as

3 $${{\varvec{\Phi}}}_{a}^{g} = \left( {{{\varvec{\Phi}}}_{a}^{T} {{\varvec{\Phi}}}_{a} } \right)^{ - 1} {{\varvec{\Phi}}}_{a}^{T}$$

As a result, the system matrices of the reduced system are ${\tilde{\mathbf{M}}}$ = **T**^T^**MT**, ${\tilde{\mathbf{K}}}$ = **T**^T^**KT** and ${\tilde{\mathbf{C}}}$ = **T**^T^**CT**, while the forcing term becomes ${\tilde{\mathbf{F}}}$ = **-T**^T^**Mτ** ${\ddot{\text{u}}}_{{\text{g}}}$. Since the optimization procedure requires an inversion of the transmission matrix **T** for each frequency interval, the SEREP technique also contributes to the reduction in the run time of the optimization algorithm.

### Modelling of nonlinear devices

The nonlinear devices utilized in this work are steel wire ropes, schematically depicted in Fig. 2b. Steel wire ropes are a commonly used solution in seismic engineering due to their dissipative behaviour. Moreover, they represent an inexpensive solution in terms of both production and maintenance costs. Thus, many researchers have investigated the hysteretic characteristics of wire ropes when subjected to shear forces^9–11^. In this context, steel wire ropes have already been successfully utilized in metafoundations. In some research works^6,27^, the goal was the protection of tanks against both horizontal and vertical ground accelerations by means of finite lattices equipped with nonlinear devices endowed with significant flexibility and hysteretic damping. Furthermore, steel wire ropes allow effective motion of the resonators along X, Y and Z directions, which can be easily deduced by Fig. 1.

To model the nonlinear dissipative behaviour of wire ropes, we employ the Bouc–Wen model. This model has been adopted to capture the hysteretic behaviours of many other seismic devices^9,10,28^.

For the sake of clarity, let us consider a single-degree-of-freedom (SDoF) system:

4 $${\rm m}\ddot{\rm u}(\rm t) + {\rm c}\dot{\rm u}(\rm t) + {\rm R}\left( {\rm t} \right) = {\rm F}({\rm t})$$

where *R*(*t*) defines the nonlinear restoring force:

5 $${\text{R}}\left( {\text{t}} \right) = \alpha \;{\text{k}}\;{\text{u}}\left( {\text{t}} \right) + \left( {1 - \alpha } \right){\text{k}}\;{\text{u}}_{{\text{y}}} \;{\text{z}}\left( {\text{t}} \right)$$

where *k* represents the yielding stiffness and *u*_*y*_ is the yielding displacement. The *z* term is a dimensionless hysteretic component provided by the solution of the following nonlinear differential equation that contains three state variables:

6 $$\dot{\rm z}\left( {\text{t}} \right) = {\text{u}}_{{\text{y}}} ^{{ - 1}} \left[ {{\text{A}}\dot{\rm u}\left( {\text{t}} \right)\; - \gamma \left| {\dot{\rm u}\left( {\text{t}} \right)} \right|\left| {{\text{z}}\left( {\text{t}} \right)} \right|^{{{\text{n}} - 1}} {\text{z}}\left( {\text{t}} \right) - \beta \dot{\rm u}\left( {\text{t}} \right)\left| {{\text{z}}\left( {\text{t}} \right)} \right|^{{\text{n}}} } \right]$$

The shape and smoothness of each hysteretic loop is controlled by the parameters *A*, *β*, *γ* and *n*. Moreover, the term *α* = *k*_*p*_/*k*_0_ in Eq. (5) defines the ratio of the postyielding stiffness to the preyielding stiffness, with

7 $${\text{k}}_{0} = \left( {\frac{{\partial {\text{R}}\left( {{\text{u}},\;\dot{\rm u},\;{\text{z}}} \right)}}{{\partial {\text{u}}}}} \right)_{{{\text{z}} = 0}} = \alpha {\text{k}} + \left( {1 - \alpha } \right){\text{kA}};\quad {\text{k}}_{{\text{p}}} = \left( {\frac{{\partial {\text{R}}\left( {{\text{u}},\;\dot{\rm u},\;{\text{z}}} \right)}}{{\partial {\text{u}}}}} \right)_{{{\text{z = z}}_{{{\text{max}}}} }} = \alpha {\text{k}}_{0}$$

and *z*_*max*_ = [*A*/(*β* + *γ*)]^1/n^.

Suitable values of *A*, *β*, *γ* and *n* govern the hardening or softening nonlinearities in the Bouc–Wen model. For instance, with |*γ*| >|*β*|, *γ* < 0, a hardening behaviour is obtained. Moreover, the elastoplastic hysteresis case is approached when *n* → *∞,* where *n* modulates the sharpness of the yield. A choice of *n* = 1 entails a closed-form solution of Eq. (6) with simple exponential functions^28^.

To identify the relevant mechanical characteristics, Paolacci and Giannini carried out an ad hoc experimental campaign^9^. On that database, we initialize the main parameters of the Bouc–Wen model. We select wire rope WR36-400-08, whose geometric dimensions are described in Table 1. In particular, *k*_0_ is the initial shear stiffness, and *R*_v_ represents the vertical load-bearing capacity. The authors^9^ found *α* = 0.254 and *u*_*y*_ = 2.2 mm.

**Table 1:** Geometric and mechanical properties of the wire ropes.

Geometric characteristics				Parameters of the Bouc–Wen model					
H [mm]	W [mm]	L [mm]	Φ [mm]	k_0_ [kN/mm]	R_y_ [kN]	u_y_ [mm]	n [–]	A [–]	α [–]
178	216	520.7	26.6	1.35	2.97	2.2	1.0	1.0	0.254

**Figure 4:**
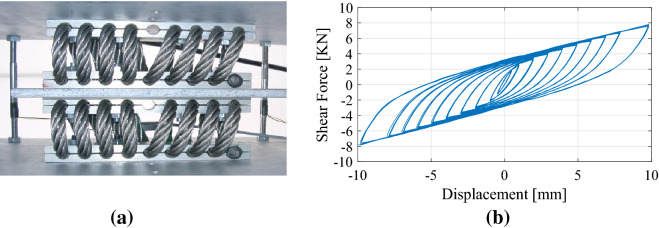
(**a**) Two wire ropes with a central plate subjected to simple shear; (**b**) hysteretic response of the wire rope under cyclic shear loading after Paolacci and Giannini^9^.

For the sake of clarity, two coupled wire ropes can be observed in the test rig of Fig. 4a; this arrangement allows cyclic simple shear tests to be reproduced by means of a central plate^9^. The corresponding hysteretic response is depicted in Fig. 4b.

**Figure 5:**
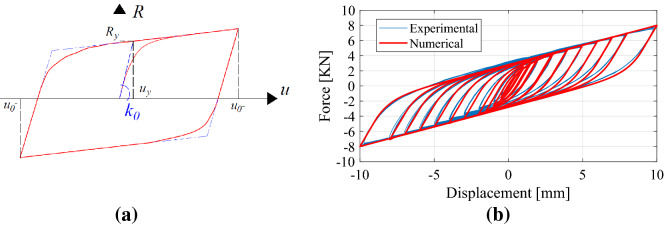
(**a**) Typical hysteretic loop of a Bouc–Wen model; (**b**) comparison between experimental and numerical responses after Paolacci and Giannini^9^.

A generic cycle of the Bouc–Wen model is shown in Fig. 5a with the main mechanical and kinematic parameters. A careful reader will notice that this model is symmetric, as can be understood from Eq. (6); nonetheless, this property does not limit its capability to properly trace the experimental response depicted both in Figs. 4b and 5b.

Note that after choosing steel wire rope, the Bouc–Wen model parameters *A* and (*β* + *γ*) can be functionally selected to be *A* = 1 and *β* + *γ* = 1. As shown by Constantinou and Adnane^29^, this choice leads to the collapse of the model to Ozdemir’s model, which is a rate-dependent Maxwell model with a nonlinear dashpot. By setting *A* = 1 in Eq. (7), indeed, the value of the initial stiffness *k* = *R*_*y*_/*u*_*y*_ = *k*_0_ is retrieved; then, by setting *β* + *γ* = 1, the maximum strength factor *z*_*max*_ in Eq. (6) becomes *z*_*max*_ = [*A*/(*β* + *γ*)]^1/*n*^ = 1 with *z* ∈ [− 1, 1].

### Seismic input model

Given the epistemic and mainly aleatoric uncertainties of the seismic input, it is not feasible to optimize a system endowed with nonlinear devices on a conventional time basis. A more accurate probabilistic (stochastic) approach is required in which both the excitation and the response are described in terms of statistical parameters such as the mean square and the variance of the vibration amplitudes. As a result, our approach relies heavily on random vibrations treated in the frequency domain, and stochastic linearization carried out in the next subsection. Therefore, the input is assumed to be a weakly stationary Gaussian-filtered white noise random process with zero mean and spectral intensity S_0_. Consequently, the soil is approximately taken into account by means of the Kanai–Tajimi filter^24^, and to avoid unrealistic high values of the excitation in the low-frequency range, we utilize an additional filter suggested by Clough and Penzien^25^. The result is the so-called Kanai–Tajimi Clough–Penzien (KTCP) filter, and the resulting PSD function is

8 $${\rm S}_{{\ddot{\rm u}_{\rm g} }} ({\upomega }) = {\rm S}_{0} \;\frac{{4\upzeta_{\rm g}^{2} \upomega_{\rm g}^{2} \upomega^{2} + \upomega_{\rm g}^{4} }}{{4\upzeta_{\rm g}^{2} \upomega_{\rm g}^{2} \upomega^{2} + \left( {\upomega_{\rm g}^{2} - \upomega^{2} } \right)^{2} }}\;\;\frac{{\upomega^{4} }}{{4\upzeta_{\rm f}^{2} \upomega_{\rm f}^{2} \upomega^{2} + \left( {\upomega_{\rm f}^{2} - \upomega^{2} } \right)^{2} }}$$

where *ω*_*g*_ = 14 rad/s is the frequency associated with the ground and *ζ*_*g*_ = 0.6 is the relevant damping ratio. The parameters *ω*_*f*_ = 0.75 rad/s and *ζ*_*f*_ = 1.9 are assumed for the low pass filter^25^, whilst the PSD intensity *S*_0_ = 0.09 m^2^/s^3^ for safe shutdown earthquakes (SSEs) corresponds to seismic activity with a return period of 2475 years. In the time domain, the KTCP filter becomes

9 $$\ddot{\rm u}_{\rm g} = \upomega_{\rm g}^{2} u_{\rm g} + 2\upzeta_{\rm g} \upomega _{\rm g} \dot{\rm u}_{\rm g} - \upomega_{\rm f}^{2} u_{\rm f} - 2\upzeta_{\rm f} \upomega_{\rm f} \dot{\rm u}_{\rm f}$$

where some variables can be treated in a state-space formulation:

10a $$\begin{aligned} \ddot{\rm u}_{\rm g} & = {\mathbf{a}}_{\rm f}^{\rm T} \,{\mathbf{u}}_{\rm f} \\ {\dot{\mathbf{\rm u}}}_{\rm f} & = {\mathbf{A}}_{\rm f} \,{\mathbf{u}}_{\rm f} + {\mathbf{V}}_{\rm f} \;{\text{ f}}({\rm t}) \\ \end{aligned}$$

10b $${\mathbf{u}}_{\rm f} = \left[ {\begin{array}{*{20}c} {{\rm u}_{\rm f} } \\ {\dot{\rm u}_{\rm f} } \\ {{\rm u}_{\rm g} } \\ {\dot{\rm u}_{\rm g} } \\ \end{array} } \right];{\mathbf{a}}_{\rm f} = \left[ {\begin{array}{*{20}c} { - \upomega_{\rm f}^{2} } \\ { - 2\upzeta_{\rm f} \upomega_{\rm f} } \\ {\upomega_{\rm g}^{2} } \\ { - 2\upzeta_{\rm g} \upomega_{\rm g} } \\ \end{array} } \right];{\mathbf{A}}_{\rm f} = \left[ {\begin{array}{*{20}c} 0 & 1 & 0 & 0 \\ { - \upomega_{\rm f}^{2} } & { - 2\upzeta_{\rm f} \upomega_{\rm f} } & {\upomega_{\rm g}^{2} } & {2\upzeta_{\rm g} \upomega_{\rm g} } \\ 0 & 0 & 0 & 1 \\ 0 & 0 & { - \upomega_{\rm g}^{2} } & { - 2\upzeta_{\rm g} \upomega_{\rm g} } \\ \end{array} } \right];{\mathbf{V}}_{\rm f} = \left[ {\begin{array}{*{20}c} 0 \\ 0 \\ 0 \\ 1 \\ \end{array} } \right]$$

where *f*(*t*) denotes the bedrock Gaussian zero-mean white noise process. Both the filter parameters and the PSD intensity *S*_0_ are chosen to fit the stationary PSD spectra of 12 natural seismic records (see Table 2) selected from Italian and European databases with a 2% probability of exceedance in 50 years.

**Table 2:** Selected natural accelerograms.

Event	Country	R, distance [km]	M, magnitude
Victoria Mexico	Mexico	13.8	6.33
Loma Prieta	USA	3.85	6.93
Northridge‐01	USA	20.11	6.69
Montenegro	Montenegro	25.00	6.90
Erzincan	Turkey	13.00	6.60
South Iceland	Island	7.00	6.50
L'Aquila Mainshock	Italy	4.87	6.30
Loma Prieta	USA	11.03	6.93
Landers	USA	11.03	7.28
South Iceland	Island	11.00	6.40
L'Aquila Mainshock	Italy	4.63	6.30
L'Aquila Mainshock	Italy	4.39	6.30

**Figure 6:**
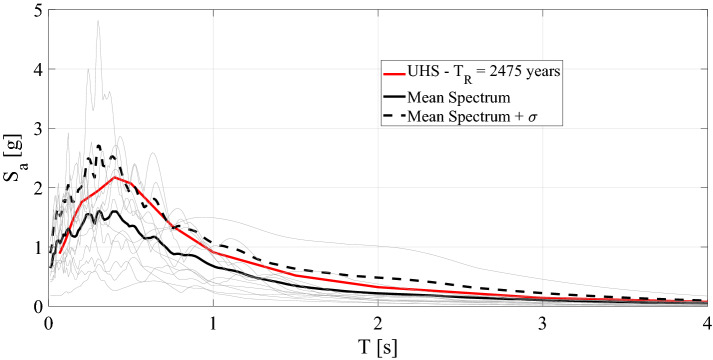
Response spectra of the selected accelerograms for SSEs; the UHS of Priolo Gargallo (Italy) is depicted in red.

The corresponding elastic response spectra, their mean and their mean plus one standard variation, together with the target uniform hazard spectrum (UHS) relevant to Priolo Gargallo, are plotted in Fig. 6. The twelve records collected in Table 2 are selected as follows. Let **s**_**0**_ be the target spectrum value vector, i.e., the mean or the mean plus one standard deviation, let ***S*** be the spectra matrix of the *n*_*a*_ accelerograms, and let *α* be the vector of the *n*_*a*_ × *1* selection coefficients *α*_*i*_. We seek for the vector α that satisfies

11 $$\left\| {\frac{1}{{\sum\limits_{i = 1}^{{n_{a} }} {\alpha_{i} } }}{\mathbf{S}}\alpha - {\mathbf{s}}_{0} } \right\|^{2} = \min$$

where $$0 \le \alpha_{i} \le 1\,$$ and $$\sum\limits_{i = 1}^{{n_{a} }} {\alpha_{i} } = n.$$

Hence, the selection is performed with all possible combinations of the *n* accelerograms among a set of *n*_*a*_ records. Thus, we can easily take into account the dispersion of the records about the mean spectrum. The discrepancy between the maximum peak of the two spectra, i.e. the UHS and the Mean Spectrum + σ should entail limited effects on the responses of the nonlinear coupled systems presented in the section *Results*.

### Equivalent linearization and optimization procedure

To model the wire ropes illustrated in Fig. 2b, which are not easily treated mathematically, we propose the stochastic linearization technique^21^. As a result, the treatment of the linearized metafoundation-tank coupled system allows the optimization procedure to be performed in the frequency domain and bypasses several difficulties related to the determination of the dispersion properties of fully nonlinear periodic systems^15^. With regard to the parameters to be optimized, to maximize antiresonance or negative effects, the masses of the resonators are set as the largest masses compatible with unit cell dimensions. Moreover, the other parameters are derived from construction or design constraints, e.g., the design column size and slab thickness; therefore, mainly the stiffness and damping of wire ropes, i.e., the relevant dissipated energy controlled by the parameters of Eqs. (5) and (6), can be optimized.

Since the system of EOMs in Eq. (1) characterizes a coupled nonlinear system, classic linear random vibration theory is not applicable. Therefore, to linearize the vector u_y_**K**^NL^**z**(t), we employ the ELT. For the sake of clarity, for an SDoF system with *N* = *1*, Eq. (6) becomes

12 $$\dot{z} + c_{eq} \dot{u} + k_{eq} z = 0$$

where *c*_*eq*_ and *k*_*eq*_ are linearization coefficients that are “equivalent” in a statistical sense^30,31^. At this stage, it is useful to introduce a state-space formulation of Eqs. (1) and (12):

13 $$\frac{d}{dt}{\mathbf{Y}} = {\mathbf{G}}\;{\mathbf{Y}} + {\mathbf{V}}\;f(t)$$

with

14 $${\mathbf{Y}}\; = \left[ {\begin{array}{*{20}c} {\mathbf{u}} \\ {{\dot{\mathbf{u}}}} \\ {\mathbf{z}} \\ {{\mathbf{u}}_{f} } \\ \end{array} } \right];{\mathbf{G}}\; = \left[ {\begin{array}{*{20}c} {{\mathbf{0}}_{{N{\text{x}}N}} } & {{\mathbf{I}}_{{N{\text{x}}N}} } & {{\mathbf{0}}_{{N{\text{x}}N}} } & {{\mathbf{0}}_{{N{\text{x}}r}} } \\ { - {\mathbf{M}}^{ - 1} \;{\mathbf{K}}^{L} } & { - {\mathbf{M}}^{ - 1} \;{\mathbf{C}}} & { - {\mathbf{M}}^{ - 1} \;{\mathbf{K}}^{NL} } & { - {\mathbf{1}}\;{\mathbf{a}}_{f}^{T} } \\ {{\mathbf{0}}_{{N{\text{x}}N}} } & { - {\mathbf{c}}_{eq} } & { - {\mathbf{k}}_{eq} } & {{\mathbf{0}}_{{N{\text{x}}r}} } \\ {{\mathbf{0}}_{{r{\text{x}}N}} } & {{\mathbf{0}}_{{r{\text{x}}N}} } & {{\mathbf{0}}_{{r{\text{x}}N}} } & {{\mathbf{A}}_{f} } \\ \end{array} } \right];{\mathbf{V}}\; = \left[ {\begin{array}{*{20}c} {{\mathbf{0}}_{{N{\text{x1}}}} } \\ {{\mathbf{0}}_{{N{\text{x1}}}} } \\ {{\mathbf{0}}_{{N{\text{x1}}}} } \\ {{\mathbf{V}}_{f} } \\ \end{array} } \right]$$

where **Y** is the state-space vector, **K**^*L*^ and **K**^*NL*^ define the linear and nonlinear components of the stiffness matrix, respectively, and **k**_eq_ and **c**_eq_ represent matrices including equivalent linear coefficients. Moreover, *N* defines the number of DoFs of the system, and *r* = 4 defines the number of equations of the KTCP filter introduced in the previous subsection. Let the covariance matrix of **Y** be **S** with *S*_*ij*_ = *E*[*y*_*i*_* y*_*j*_], and assume that the seismic input is stationary. The solution of Eq. (13) can be derived from the following Lyapunov system of equations:

15 $${\mathbf{G}}\;{\mathbf{S}} + {\mathbf{S}}\;{\mathbf{G}}^{T} + {\mathbf{B}} = {\mathbf{0}}$$

where **B** is a zero matrix except for the generic diagonal element corresponding to the nonzero row of the forcing function vector, i.e., B_ij_ = 2πS_0_. Equation (15) is solved with the algorithm proposed by Bartels and Steward^32^. As expected, **k**_eq_ and **c**_eq_ are not known a priori; in this regard, Maldonado et al.^30^ suggested setting the initial values for an iterative solution procedure *c*_*eq*_ = 1 and *k*_*eq*_ = 0.05 (*β* + *γ*) for faster convergence. Further details about the whole procedure are available in Maldonado et al.^30^ and Spanos et al.^31^.

To operate in the frequency domain, we start from Eq. (4) for a SDoF system and define the transfer function **H**(ω) of the coupled system depicted in Fig. 3. However, Eq. (13) includes the KTCP filter, and the derivation is more burdensome for an MDoF system. Therefore, the relevant H(ω) becomes

16 $${\text{H}}\left( \omega \right) = \left[ { - \omega^{2} m + i\omega c + \alpha k - \frac{i\omega }{{i\omega + k_{eq} }}c_{eq} \left( {1 - \alpha } \right)ku_{y} } \right]^{ - 1}$$

where the details of the derivation can be found in the Supplementary Material. Its generalization is expressed as

17 $${\mathbf{H}}\left( \omega \right) = \left[ { - \omega^{2} {\mathbf{M}} + i\omega \;{\mathbf{C}} + {\mathbf{K}}\; + \;{\mathbf{K}}^{{{\text{eq}}}} } \right]^{ - 1}$$

where ${\mathbf{K}}^{{{\text{eq}}}}$ contains zero terms except those in which the *n*th resonator is physically connected. More precisely, the nonzero terms $k_{ij}^{eq}$ of matrix ${\mathbf{K}}^{{{\text{eq}}}}$ are

18 $$k_{ij}^{eq} = \; - \frac{i\omega }{{i\omega + k_{eq} }}c_{eq} \left( {1 - \alpha_{n} } \right)u_{y\;} k_{n}$$

where *α*_*n*_ and *k*_*n*_ refer to the *n*th resonator of the metafoundation. Note that for *α* = 1, the transmission matrix in Eq. (17) degenerates into a linear transmission matrix.

To carry out the optimization, we minimize the interstorey displacement and the absolute acceleration of the tank’s impulsive mode, where the relevant variances σ_dr_ and σ_acc_, respectively, are expressed as

19 $$\begin{aligned} \sigma_{dr}^{2} & = \int\limits_{0}^{ + \infty } {\left| {{\text{H}}_{imp} \left( \omega \right) - {\text{H}}_{res} \left( \omega \right)} \right|^{2} S_{{\ddot{u}_{g} }} \left( \omega \right)d\omega } \\ \sigma_{acc}^{2} & = \int\limits_{0}^{ + \infty } {\left| {1 - \omega^{2} {\text{H}}_{imp} \left( \omega \right)} \right|^{2} S_{{\ddot{u}_{g} }} \left( \omega \right)d\omega } \\ \end{aligned}$$

where *H*_*imp*_(*ω*) defines the transfer function of the impulsive mass and *H*_*res*_(*ω*) is the transfer function of the resonator’s layer. The dimensionless performance indices are defined as follows:

20 $$\begin{aligned} {\text{PI}}_{{{\text{dr}}}} & = \frac{{\sigma_{dr}^{2} }}{{\sigma_{dr,fixed}^{2} }} \\ {\text{PI}}_{{{\text{acc}}}} & = \frac{{\sigma_{acc}^{2} }}{{\sigma_{acc,fixed}^{2} }} \\ \end{aligned}$$

where $\sigma_{dr,fixed}^{2}$ and $\sigma_{acc,fixed}^{2}$ represent the variances of the interstorey drift and the absolute acceleration, respectively, w.r.t. a clamped tank.

The optimization procedure relies on the design variables *k*_*k,n*_ and *β*_*k,n*_ collected in the parameter vector **X**^NL^:

21 $${\mathbf{X}}^{{\text{NL}}} = \left[ {k_{1,1} ,\;k_{k,n} ,\;A_{1,1} ,\;A_{k,n} ,\;\beta_{1,1} ,\;\beta_{k,n} ,\;\gamma_{1,1} ,\;\gamma_{k,n} } \right]^{\text{T}}$$

The statement of our optimization problem is as follows:

22 $$\mathop {\min }\limits_{k,n} \;\;{\text{PI}}_{dr} \left( {{\mathbf{X}}^{{\text{NL}}} } \right)\;{\rm or}\;\mathop {\min }\limits_{k,n} \;\;{\text{PI}}_{acc} \left( {{\mathbf{X}}^{{\text{NL}}} } \right)$$

where *k* = (1,…,*n*_*k*_) and *n* = (1,…,*n*_*r*_). The limits imposed on the design variable *β*_*k,n*_ are

23 $$0 < {\beta_{k,n}} < 1$$

Further details about the bound of Eq. (23) were already provided in the section *Modelling of nonlinear devices*.

### Dispersion characteristics of the linearized periodic system

Though the system is linearized by means of Eq. (12), the effects of the nonlinear devices depicted in Fig. 2 on the band structure of the relevant periodic system are of interest. On the one hand, we can observe the effect of equivalent damping on the dispersion relationships; on the other hand, we can appreciate the effects of the PSD *S*_0_.

**Figure 7:**
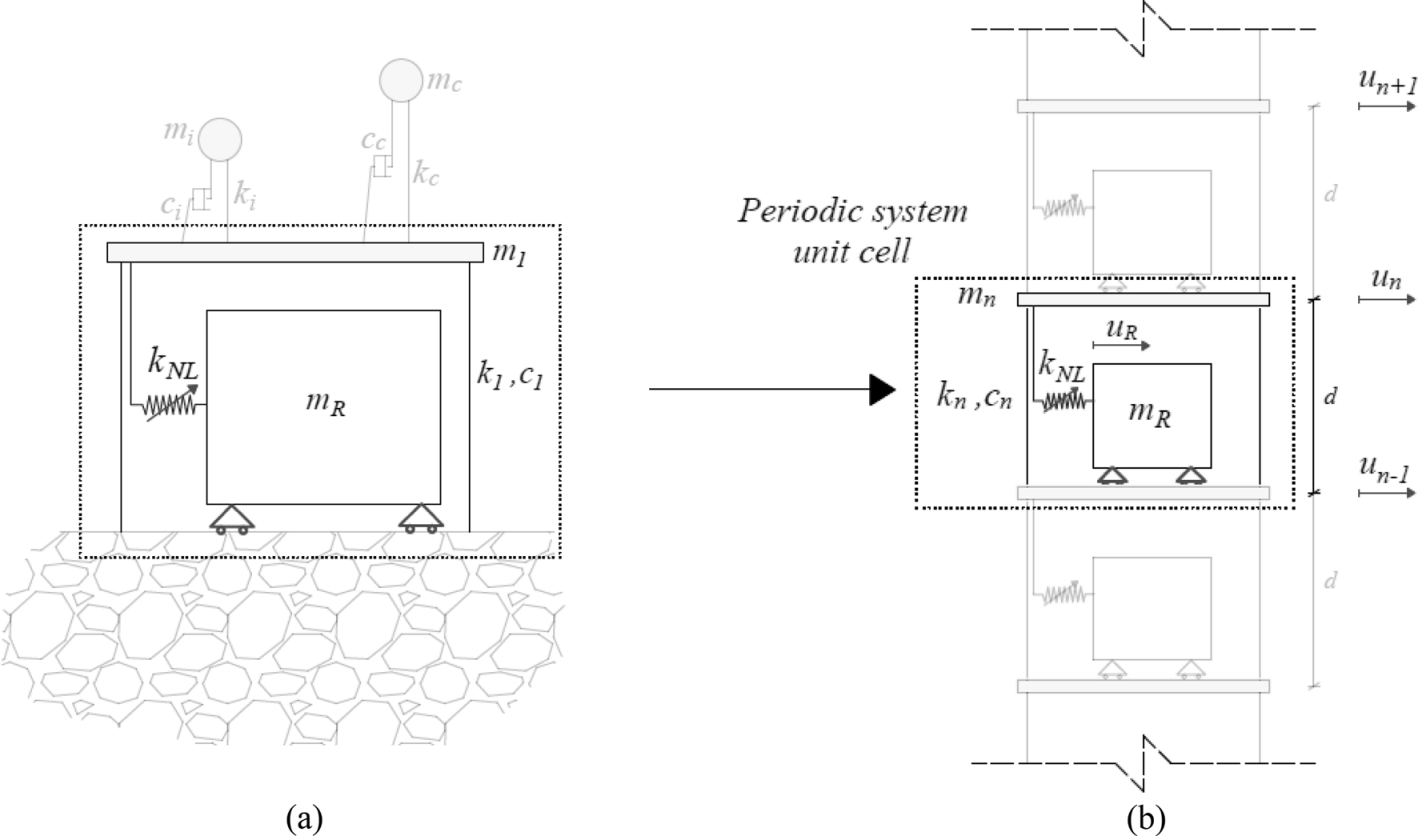
Dynamic systems: (**a**) coupled finite lattice metafoundation-tank system; (**b**) metafoundation modelled as a periodic system.

First, let us consider Fig. 7, which shows both the finite lattice 1-layer structure (see Fig. 7a) and the relevant system of repetitive unit cells depicted in Fig. 7b.

The 2-DoF system associated with the unit cell, i.e., slab and resonator of Fig. 7b, can be represented as follows:

24 $$\left\{ {\begin{array}{*{20}l} {{\rm m}_{\rm n} \ddot{\rm u}_{\rm n} + {\rm c}_{\rm n} (\dot{\rm u}_{\rm n} - \dot{\rm u}_{{\rm n}- 1} ) + {\rm c}_{\rm n} (\dot{\rm u}_{\rm n} - \dot{\rm u}_{{\rm n}+ 1} ) + {\rm k}_{\rm n} ({\rm u}_{\rm n} - {\rm u}_{{\rm n}- 1} ) + {\rm k}_{\rm n} ({\rm u}_{\rm n} - {\rm u}_{{\rm n}+ 1} ) - \alpha {\rm k}_{0} ({\rm u}_{\rm R} - {\rm u}_{\rm n} ) - (1 - \alpha ){\rm k}_{0} {\rm u}_{\rm y} {\text{z(t)}} = 0} \hfill \\ {{\rm m}_{\rm R} \ddot{\rm u}_{\rm R} + \alpha {\rm k}_{0} ({\rm u}_{\rm R} - {\rm u}_{\rm n} ) + (1 - \alpha ){\rm k}_{0} {\rm u}_{\rm y} {\text{z(t)}} = 0} \hfill \\ \end{array} } \right.$$

where dots refer to time differentiation. We assume the following harmonic solution for displacements:

25 $$\begin{aligned} {\rm u}_{\rm n} & = \tilde{\rm u}_{\rm n} {\rm e}^{{\rm i}\omega {\rm t}} \\ {\rm u}_{\rm R} & = \tilde{\rm u}_{\rm R} {\rm e}^{{\rm i}\omega {\rm t}} \\ \end{aligned}$$

where ũ_n_ and ũ_R_ are displacement amplitudes. In addition, the Floquet-Bloch theorem can be applied as

26 $$\begin{aligned} {\rm u}_{{\rm n} + 1} & = \tilde{\rm u}_{\rm n} {\rm e}^{{\rm i}\mu } \\ {\rm u}_{{\rm n} - 1} & = \tilde{\rm u}_{\rm n} {\rm e}^{ - {\rm i}\mu } \\ \end{aligned}$$

where *μ* = *κd* defines the so-called propagation constant based on the wavenumber *κ* and the unit cell length *d*. Thus, the application of the ELT by means of Eqs. (12) and (18) and the conditions of Eqs. (25)–(26) to the system of Eq. (24) entails the following:

27 $$\left\{ {\begin{array}{*{20}l} {\left[ { - \omega ^{2} {\text{m}}_{{\text{n}}} + 2{\text{k}}_{{\text{n}}} + 2{\text{i}}\omega {\text{c}}_{{\text{n}}} + \alpha {\text{k}}_{0} - \frac{{{\text{i}}\omega }}{{{\text{i}}\omega + {\text{k}}_{{{\text{eq}}}} }}{\text{c}}_{{{\text{eq}}}} (1 - \alpha ){\text{k}}_{0} {\text{u}}_{{\text{y}}} } \right]{\text{u}}_{{\text{n}}} + \left[ { - \alpha {\text{k}}_{0} + \frac{{{\text{i}}\omega }}{{{\text{i}}\omega + {\text{k}}_{{{\text{eq}}}} }}{\text{c}}_{{{\text{eq}}}} (1 - \alpha ){\text{k}}_{0} {\text{u}}_{{\text{y}}} } \right]{\text{u}}_{{\text{R}}} - ({\text{k}}_{{\text{n}}} + {\text{i}}\omega {\text{c}}_{{\text{n}}} )({\text{e}}^{{ - {\text{i}}\mu }} + {\text{e}}^{{{\text{i}}\mu }} ){\text{u}}_{{\text{n}}} = 0} \hfill \\ {{\text{u}}_{{\text{R}}} = \frac{{\alpha {\text{k}}_{0} - \frac{{{\text{i}}\omega }}{{{\text{i}}\omega + {\text{k}}_{{{\text{eq}}}} }}{\text{c}}_{{{\text{eq}}}} (1 - \alpha ){\text{k}}_{0} {\text{u}}_{{\text{y}}} }}{{ - \omega ^{2} {\text{m}}_{{\text{n}}} + \alpha {\text{k}}_{0} - \frac{{{\text{i}}\omega }}{{{\text{i}}\omega + {\text{k}}_{{{\text{eq}}}} }}{\text{c}}_{{{\text{eq}}}} (1 - \alpha ){\text{k}}_{0} {\text{u}}_{{\text{y}}} }}{\text{u}}_{{\text{n}}} } \hfill \\ \end{array} } \right.$$

The substitution of u_R_ into the first equation of Eq. (27) leads to the following dispersion relation:

28 $$\mu = {\text{arccos}}\left( {\frac{{ - \omega ^{2} {\text{m}}_{{\text{n}}} + 2{\text{k}}_{{\text{n}}} + 2{\text{i}}\omega {\text{c}}_{{\text{n}}} + \alpha {\text{k}}_{0} - \frac{{{\text{i}}\omega }}{{{\text{i}}\omega + {\text{k}}_{{{\text{eq}}}} }}{\text{c}}_{{eq}} (1 - \alpha ){\text{k}}_{0} {\text{u}}_{{\text{y}}} + \frac{{\left[ { - \alpha ^{2} {\text{k}}_{0}^{2} + 2\alpha {\text{k}}_{0} \frac{{{\text{i}}\omega }}{{{\text{i}}\omega + {\text{k}}_{{{\text{eq}}}} }}{\text{c}}_{{{\text{eq}}}} (1 - \alpha ){\text{k}}_{0} {\text{u}}_{{\text{y}}} - \left( {\frac{{{\text{i}}\omega }}{{{\text{i}}\omega + {\text{k}}_{{{\text{eq}}}} }}{\text{c}}_{{{\text{eq}}}} (1 - \alpha ){\text{k}}_{0} {\text{u}}_{{\text{y}}} } \right)^{2} } \right]}}{{ - \omega ^{2} {\text{m}}_{{\text{R}}} + \alpha {\text{k}}_{0} - \frac{{{\text{i}}\omega }}{{{\text{i}}\omega + {\text{k}}_{{{\text{eq}}}} }}{\text{c}}_{{{\text{eq}}}} (1 - \alpha ){\text{k}}_{0} {\text{u}}_{{\text{y}}} }}}}{{2({\text{k}}_{{\text{n}}} + {\text{i}}\omega {\text{c}}_{{\text{n}}} )}}} \right)$$

in the (*μ*-*ω*) plane.

## Results

### Optimization and time-history analysis results

**Figure 8:**
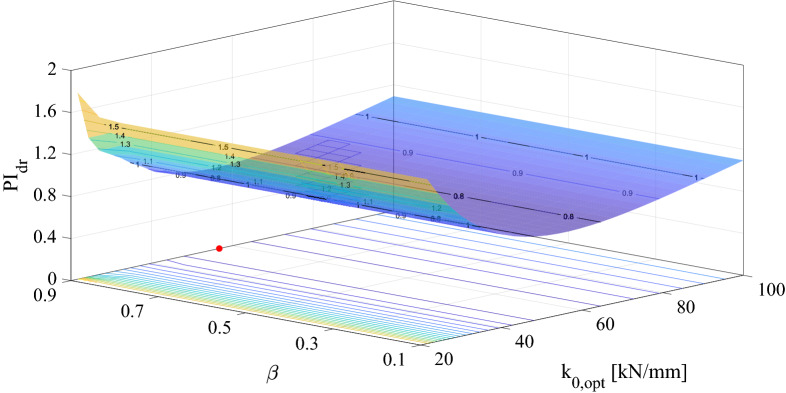
Optimal surface for the linearized CMS.

The optimization and time history analyses based on the methods discussed at length in the previous section are presented and discussed herein. We set *n*, *α* and *u*_*y*_ based on the properties described in Table 1, and we search for the optimal values of *k*, *β* and *γ* with the constraints^29^ *A* = 1 and *β* + *γ* = 1. The results of the optimization process based on the index PI_*dr*_ introduced in Eq. (20) are depicted in Fig. 8 for the CMS model. The minimum value of *PI*_*dr*_ = 0.78, i.e., the red point on the *β* − *k*_0,*opt*_ plane, corresponds to *k*_0,*opt*_ = 56.8 KN/mm and *β*^*opt*^ = 0.9. *β* and *γ* quantify the dissipation characteristics of wire ropes, and with *β* + *γ* = 1, *γ*^*opt*^ = 0.1. Notably, *k*_0,*opt*_ corresponds to the horizontal stiffness of a single resonator of the metafoundation. Similar values can be obtained by means of the index *PI*_*acc*_.

**Figure 9:**
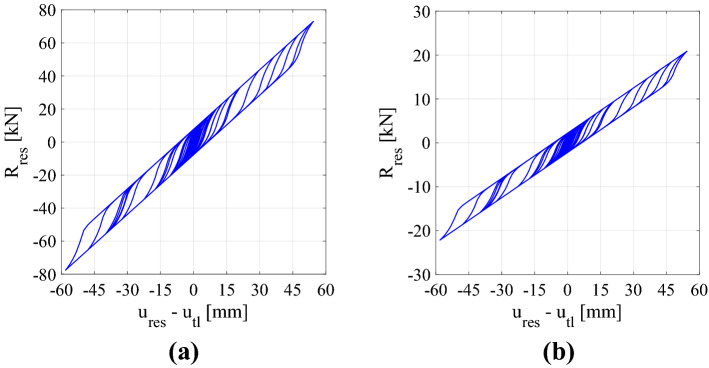
Hysteretic loops of the one-layer CMS hysteretic damper for *A* = 1, *β* = 0.9 and *γ* = 0.1. The resonators are equipped with the (**a**) optimal and (**b**) minimum numbers of wire ropes.

To test the performance of the metafoundation-tank coupled system, THAs are carried out considering the hysteretic responses of the devices in agreement with Eq. (5). Figure 9 shows the hysteretic loops of a single wire rope when the CMS is subjected to one of the 12 accelerograms listed in Table 2. The term *u*_*res*_ represents the displacement of the generic resonator w.r.t. *u*_*tl*_, i.e., the displacement of the top of the metafoundation. Figure 9a refers to the optimized system where 42 wire ropes per resonator are needed. Conversely, Fig. 9b refers to the system provided with the minimum number of wire ropes required to support a resonator, i.e., 16 wire ropes.

**Figure 10:**
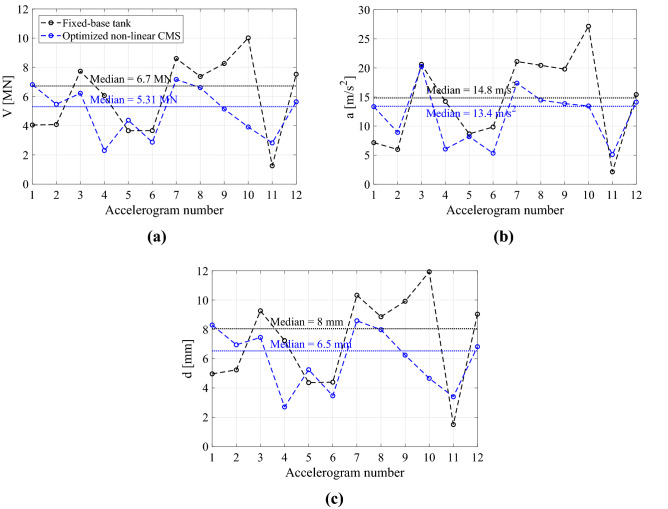
Maximum and median values of the (**a**) base shear, (**b**) absolute acceleration and (**c**) interstorey drift of the optimized nonlinear CMS w.r.t. the fixed-base tank for each accelerogram listed in Table 2.

To appreciate the performance of the optimized finite lattice metafoundation, Fig. 10 depicts both the maximum and the median parameter values of the nonlinear foundation-tank coupled system w.r.t. the fixed-base solution when subjected to the 12 seismic records listed in Table 2. The maximum values of the base shear *V*, absolute acceleration *a* and interstorey drift *d* of the impulsive mass are reported. Nonlinear devices are characterized by *β* and *γ* equal to 0.9 and 0.1, respectively. The favourable performance of the metafoundation, which achieves reductions of approximately 21%, 10% and 19% in *V*, *a*, and *d*, respectively, w.r.t. the fixed-base case, is evident.

**Figure 11:**
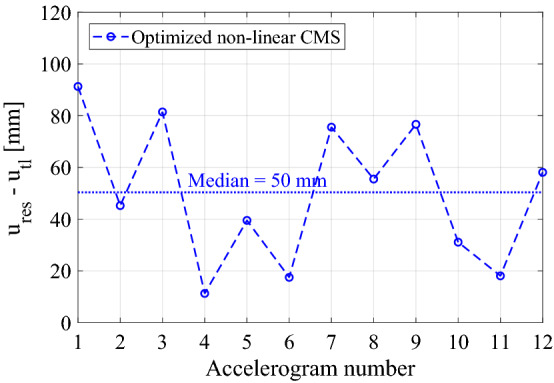
Maximum and median values of wire ropes for the optimized nonlinear CMS for each time history.

Finally, Fig. 11 shows the maximum and median values of the wire rope displacements in the optimized nonlinear CMS relevant to each time history. The maximum displacements reach approximately 90 mm with a median equal to 50 mm. These demand values are compatible with the capacities of standard wire ropes.

### Dispersion curves of the linearized periodic system

**Figure 12:**
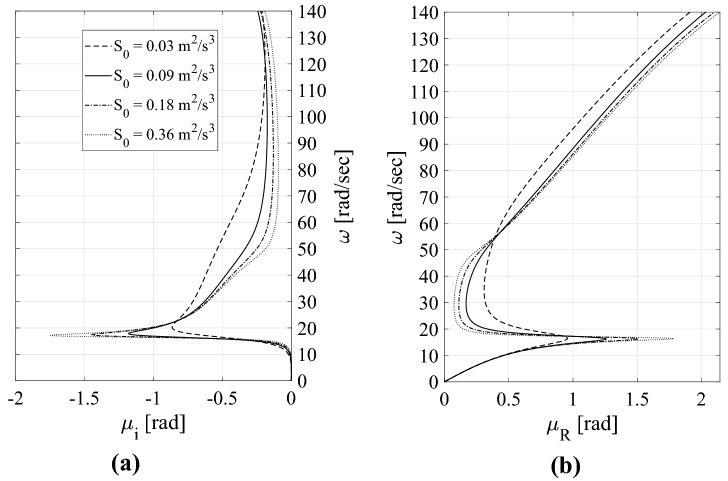
Periodic metafoundation dispersion curves: (**a**) imaginary component of *μ*; (**b**) real component of *μ*.

For the sake of completeness, it is worthwhile to examine some wave propagation properties of the decoupled metafoundation depicted in Fig. 7a and modelled as a periodic system in Fig. 7b. We rely on the ELT applied to nonlinear devices so that we are able to trace the mode shape families, i.e., the dispersion curves or band structures, defined by means of wavelengths—inversely, wavenumbers—and frequencies. As the linearized hysteretic system exhibits a significant amount of damping (see Eqs. (16) and (17)), the damped periodic materials can be characterized by means of (1) complex wavenumbers as a function of real frequencies and (2) complex frequencies as a function of real wavenumbers. We adopt the former methodology, where the dispersion relationships are obtained with Eq. (28) in terms of *μ*(*ω*)*.* This condition corresponds to a harmonic wave motion where a driving frequency *ω* is prescribed. The values of the propagation constant *μ*(*ω*) are all complex due to the presence of damping. The relevant dispersion relationships representative of the periodic metafoundation depicted in Fig. 7b are shown in Fig. 12. The relationships are expressed by real values of *μ* corresponding to the propagative index of waves, while the imaginary components of *μ*, often called the attenuation constant, define the spatial decay of the amplitude as the wave progresses through the lattice.

Each dispersion curve shown in Fig. 12 is associated with a different value of *k*_*eq*_ that, due to the linearization process, depends on the input PSD S_0_. Standard ELT results^30,31^ show that an increase in S_0_ from 0.03 to 0.36 m^2^/s^3^ entails an increase in *k*_*eq*_—83, 177, 273 and 410—whilst *c*_*eq*_ remains essentially unchanged and equal to *c*_*eq*_ =  − 269. As shown by Spanos and Giaralis^33^, *k*_*eq*_ and *c*_*eq*_ are derived from a third-order ELT whose values do not correspond to any particular mechanical system; hence, their physical significance is limited. Conversely, if we rely on a second-order statistical linearization scheme^33^ governed by the linearization parameters ζ_eq_ and ω_eq_, this leads to a trend where an increase in S_0_ corresponds to a reduction in ω_eq_, as understood from Fig. 12a. Note that for the highest value of S_0_, i.e., almost an undamped-like structure, the curve tends to create a bandgap. In that area, the *μ*_*i*_ values are higher for curves with less damping. Finally, with regard to waves travelling at frequencies that belong to the passband of *μ*_*R*_, higher damping values entail greater spatial wave attenuation.

### Numerical investigation of the periodic system

**Figure 13:**
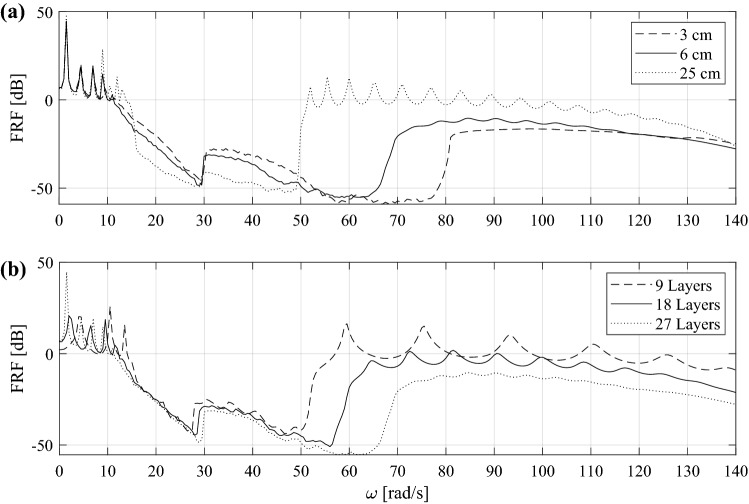
Numerical FRFs of a periodic finite lattice: (**a**) FRF for several input amplitudes *A*_0_ for 27 layers; (**b**) FRF for several layers for *A*_0_ = 6 cm.

In contrast to the previous subsection, where we considered a linearized periodic system, in this section we carry out numerical simulations on the metafoundation’s periodic configuration depicted in Fig. 7b; thus, we do not make any assumptions about the nonlinearities involved. The system is excited by a time-harmonic displacement *A*_*0*_ applied to the bottom layer. The output response *A* is read from the top layer. The boundary condition of the system of masses is free-free, and since the system is not excited by a force, rigid-body motion is avoided. The frequency response function (FRF) is then evaluated as

29 $$FRF = 20\log_{10} \frac{A}{{A_{0} }}$$

where *A* is the maximum amplitude of the steady-state response and *A*_*0*_ is the amplitude of the harmonic excitation. The resulting wave transmittances are plotted in Fig. 13.

The FRFs of Fig. 13 are clearly amplitude dependent because the system is nonlinear. However, the wave transmittances in Fig. 13a for low frequencies show that the modal resonances do not depend strongly on the amplitude excitation. This is clearly confirmed by the linearized band structure observed in Fig. 12, where the acoustic branch appears to be identical for each S_0_. Nevertheless, the identification of an optical branch is simpler for high amplitudes, i.e., *A*_0_ = 25 cm; in fact, the presence of resonance points becomes evident.

We can also identify a strong attenuation zone comparable to the band gap. The width of the band gap varies with the displacement amplitude. A wide and deep band gap can be observed under small-amplitude excitations, whereas the width and depth of the band gap are reduced by increasing the input amplitude. This leads to a worse wave attenuation performance of the hysteretic system under high-amplitude vibrations. The performance of the periodic hysteretic system can also be evaluated by means of Fig. 13b for *A*_0_ = 6 cm. It is clear that the performance level increases in terms of the width and depth of the band gap as the system is characterized by more layers. Moreover, it is worth noting that both FRFs identify an attenuation zone accurately predicted by the linearized dispersion curves of Fig. 12. In fact, the trend of the imaginary components of *μ* is centred at the resonator’s linearized frequency *ω*_*R*_ = (*αk*_0_/*m*_*R*_)^1/2^ = 16,9 rad/s. Figure 13 consistently shows that for frequencies ranging from approximately 15 to 20 rad/sec, the FRF starts to decrease. Finally, note that the stiffness *αk*_0_ corresponds to the slope of the hardening branch of the Bouc–Wen model in Eq. (7).

## Discussion

The objective of this work was to conceive a metafoundation bearing oil storage tanks capable of inheriting the filter properties of finite lattice phononic structures. More precisely, the vibrations in the frequency regime induced by seismic records are attenuated by the favourable properties of coupled hysteretic devices and resonators embedded in the metafoundation. The metafoundation is composed of massive vibrating concrete blocks that are coupled to the slabs by means of fully nonlinear hysteretic devices. Moreover, to achieve cost savings, the foundation is constituted by a single layer of resonators.

To identify the effective properties of nonlinear hysteretic devices in terms of stiffness and equivalent damping, an optimization procedure was carried out by minimizing two dimensionless performance indices based on the variances of interstorey displacement of the impulsive mass and of the relevant absolute acceleration. Because the optimization procedure has to be carried out in the frequency domain for linear time-invariant systems subjected to stationary seismic records, we applied the stochastic linearization technique^19^ to the nonlinear hysteretic devices embedded in the metafoundation. As a result, the optimized metafoundation-tank coupled system performed well when subjected to natural seismic records carried out in the time domain. In addition, we determined the dispersion relations for the periodic linearized spring–mass chain, see Fig. 7b, which clearly depends on the PSD *S*_*0*_ amplitude. As a result, the maximum attenuation rate, based on the propagation constant depicted in Fig. 12a, increases with an increase in *S*_*0*_ and a decrease in the equivalent damping *ζ*_*eq*_ in the resonators.

Furthermore, we investigated the nonlinear response of the periodic metafoundation by means of numerical FRFs. The results confirm the reliability of the dispersion analysis subsequent to applying the equivalent linearization technique. In fact, a strong attenuation zone in the FRF is located in the frequency range where the dispersion curves indicate very high values of the imaginary propagation constant *μ*_*i*_.

The results achieved herein reveal a promising application of finite lattice metafoundations to large systems subjected to strong seismic excitations. In addition, this work paves some ways: (1) the application of stochastic linearization to a finite lattice allows the metafoundation-tank coupled nonlinear system to be optimized while capturing the variability of the seismic input, and (2) the use of simple hysteretic devices such as wire ropes permits the 3D motion of massive resonators and reflect the great effectiveness and potential of these devices as vibration mitigation tools.

Ultimately, the validation of these results by means of the 3D physical characterization of hysteretic devices as well as the analysis of nonlinear wave mechanisms through nonlinear spatial analyses deserve further study.

## Supplementary Information

Supplementary Information.
