# Supplementary material for: Vibration mitigation of an MDoF system subjected to stochastic loading by means of hysteretic nonlinear locally resonant metamaterials
Source: Sci Rep. 2021 May 6;11:9728. doi: 10.1038/s41598-021-88984-0 (PMC8102642; doi:10.1038/s41598-021-88984-0)
Supplement: Supplementary file 1 — Supplementary Information. [file 41598_2021_88984_MOESM1_ESM.pdf]

Vibration mitigation of an MDoF system subjected to stochastic loading by means of hysteretic nonlinear locally resonant metamaterials

# - APPENDIX -

Francesco Basone[1,2], Oreste S. Bursi[2], Fabrizio Aloschi[2,3], Günter Fischbach[3]

[1] Engineering and Architecture Faculty, University of Enna "Kore", Viale delle Olimpiadi, 94100 Enna, Italy.
email: francescobasone@gmail.com

[2] Department of Civil, Environmental and Mechanical Engineering, University of Trento, Via Mesiano 77, 38123 Trento, Italy.
email: oreste.bursi@unitn.it; fabrizio.aloschi@unitn.it

[3] IGF - Ingenieurgesellschaft Dr. Ing. Fischbach mbH, An der Vogelrute 2, 50374 Erftstadt-Lechenich, Germany.
email: gf@ig-fischbach.de

This Section explains how the transmission matrix H(ω) is obtained for the SDoF resonator linked to the concrete slab by means of wire ropes depicted in Fig. 2(b). They were discussed in the Subsection *Modelling of nonlinear devices* of "Vibration mitigation of an MDoF system subjected to stochastic loading by means of hysteretic nonlinear locally resonant metamaterials" by Basone et al. 2021.

In order to solve Eq. (12), we consider a Cauchy problem, whose solution reads,

$$z(t) = -c_{eq} e^{-k_{eq}t} \left(\int e^{k_{eq}t} \dot{u}(t)\, dt + c \right) \tag{A.1}$$

where c defines the integration constant.

The value $c = 0$ derives from the initial conditions $z(t=0)=0$ and $\dot{u}(t=0)=0$. Then, if one replaces (A.1) in both (4) and (5) obtains,

$$m\ddot{u}(t) + c\dot{u}(t) + \alpha k u(t) + (1-\alpha) k u_y \left[-c_{eq} e^{-k_{eq}t} \int e^{k_{eq}t} \dot{u}(t)\, dt \right] = F(t) \tag{A.2}$$

The conditions $u(t) = u_0 e^{i\omega t}$ and $F(t) = F_0 e^{i\omega t}$ entail

$$-\omega^2 u_0 e^{i\omega t} m + i\omega u_0 e^{i\omega t} c + \alpha u_0 e^{i\omega t} k + (1-\alpha) k u_y \left[-c_{eq} e^{-k_{eq}t} \int i\omega\, u_0 e^{k_{eq}t} e^{i\omega t} dt \right] = F_0 e^{i\omega t} \tag{A.3}$$

As a result, the integral term in (A.3) can be solved as

$$\int i\omega\, u_0 e^{(i\omega + k_{eq})t} dt = \frac{i\omega}{i\omega + k_{eq}} u_0 e^{(i\omega + k_{eq})t} \tag{A.4}$$

and, thus, (A.3) becomes,

$$-\omega^2 u_0 e^{i\omega t} m + i\omega u_0 e^{i\omega t} c + \alpha u_0 e^{i\omega t} k - \frac{i\omega}{i\omega + k_{eq}} c_{eq} (1-\alpha) k u_y u_0 e^{i\omega t} = F_0 e^{i\omega t} \tag{A.5}$$

The transfer function H(ω) finally reads,

$$\mathrm{H}(\omega) = \left[-\omega^2 m + i\omega c + \alpha k - \frac{i\omega}{i\omega + k_{eq}} c_{eq} (1-\alpha) u_y k \right]^{-1} \tag{A.6}$$
